# The Effect of Tai Chi Chuan on Emotional Health: Potential Mechanisms and Prefrontal Cortex Hypothesis

**DOI:** 10.1155/2021/5549006

**Published:** 2021-04-30

**Authors:** Ying Yao, Likun Ge, Qian Yu, Xiaohong Du, Xiangyang Zhang, Ruth Taylor-Piliae, Gao-Xia Wei

**Affiliations:** ^1^Key Laboratory of Mental Health, Institute of Psychology, Chinese Academy of Sciences, Beijing, China; ^2^Department of Psychology, University of Chinese Academy of Sciences, Beijing, China; ^3^Department of Child Rehabilitation, The Fifth Affiliated Hospital of Zhengzhou University, Zhengzhou, Henan, China; ^4^College of Physical Education and Sports, Beijing Normal University, Beijing, China; ^5^College of Nursing, University of Arizona, Tucson, USA; ^6^Key Laboratory of Behavioral Science, Institute of Psychology, Chinese Academy of Sciences, Beijing, China

## Abstract

Deep involvement in the negative mood over long periods of time likely results in emotional disturbances/disorders and poor mental health. Tai Chi Chuan (TCC) is regarded as a typical mind-body practice combining aerobic exercise and meditation to prevent and treat negative mood. Although there are an increasing number of TCC studies examining anxiety, depression, and mental stress, the mechanisms underlying these negative emotions are not fully understood. This review study examined TCC studies related to emotional health from both clinical patients and healthy individuals. Next, several potential mechanisms from physiological, psychological, and neurological perspectives were evaluated based on direct and indirect research evidence. We reviewed recent functional magnetic resonance imaging studies, which demonstrated changes in brain anatomy and function, mainly in the prefrontal cortex, following TCC practice. Finally, the effects of TCC on emotion/mental health is depicted with a prefrontal cortex hypothesis that proposed “an immune system of the mind” indicating the role of the prefrontal cortex as a flexible hub in regulating an individual's mental health. The prefrontal cortex is likely a key biomarker among the multiple complex neural correlates to help an individual manage negative emotions/mental health. Future research is needed to examine TCC effects on mental health by examining the relationship between the executive control system (mainly prefrontal cortex) and limbic network (including amygdala, insula, and hippocampal gyrus).

## 1. Introduction

There are an estimated 350 million people of all ages worldwide, suffering from depression [1]. Similarly, anxiety disorders are among the most prevalent mental health conditions, despite differences in cultural beliefs and practices. Mental health conditions, such as depression and anxiety, are highly intertwined with the leading causes of illness and disability (e.g., heart disease and stroke), leading to high family and social burden due to increased healthcare costs. Many clinicians and research scientists support mental health prevention (instead of intervention) for treating disorders [2]. Although there are known effective treatments for mental health disorders, worldwide, fewer than half of those individuals affected (many countries are less than 10%) received any treatments due to a variety of barriers such as high costs, lack of facilities, or challenges associated with diagnosis of the condition [3]. Given the accessibility of physical activity among individuals of all ages, it is promising to develop new, simple, and effective strategies to prevent and manage mental health disorders.

Tai Chi Chuan (TCC) is one of the Chinese traditional exercises which includes the three treasures of Jing, Qi and Shen, according to Taoism philosophy and based on hundreds of years of practice [4]. It emphasizes the key roles of mind and body, the harmony and coordination of these two components, as well as the importance of simultaneous performance of relaxation and concentration. Currently, increasing evidences showed that TCC practice could significantly enhance positive emotions and alleviate depression, anxiety, as well as mental stress [4–6]. Regarding the mechanism underlying the effect of TCC on mental health, researchers have proposed several theoretical hypotheses from the features of TCC practice. Some address the traditional feature of TCC on aerobic activity, linking to increased levels of brain-derived neurotrophic factors implicated in mood disorders [7, 8]. Others suggest that the beneficial effects of TCC might be associated with breath and imagery-related changes in autonomic tone [9]. Recently, one hypothesis is provided to emphasize the role of body postures during TCC practice [4]. It demonstrates that the body shapes and movement patterns trained in TCC may be associated with the improvements in psychological well-being reported in clinical trials. Specifically, the specific static and dynamic postures including balanced muscular tone and steadier gait dynamics might exert influence on mood [10]. These assumptions undoubtedly advance the better understandings of the effects of TCC on emotional health from multiple perspectives. However, it remains largely unknown for the neurophysiological mechanism underlying the effect of TCC practice on emotional-related health outcomes [11–13]. Thus, the aim of this study was (1) to summarize the existing TCC studies reporting both negative and positive emotions impacting mental health and (2) to analyze the potential mechanisms of how TCC works to improve emotional health from physiological, psychological, and neurological perspectives.

Therefore, three English-language databases (PubMed, Web of Science, and EBSCO) and two Chinese-language databases (China National Knowledge Infrastructure and Wanfang) were searched from inception until October 2020. To obtain a maximum of relevant studies, we used three groups of keywords: “anxiety” OR “depression” OR “stress” OR “emotion” OR “affect”; “Tai Chi” OR “taiji.” A total of 400 articles were retrieved based on the searching result. We found that the number of publications is dramatically increasing, especially in the last decade (Figure S1). Finally, we added “functional MRI” OR “MRI” OR “EEG” OR “ERP” OR “fNIRS” to search the brain imaging studies relevant to this topic. Only 6 studies were retrieved including two reviews.

## 2. The Effect of TCC Practice on Emotional Health

### 2.1. The Effect of TCC Practice on Depression

Self-report measurements such as Hamilton Depression scale [14], Depression scales [15], and Beck Depression Inventory [16] are widely applied in studies on the effect of TCC on depression. A large sample cross-sectional study using the elderly depression scale and the depression self-report scale showed that the depression odds ratios were significantly reduced after TCC practice (*F* (1, 27) = 6.61, *p* < 0.05, partial *η*^2^ = 0.19) [17]. Another study found that after 8-week TCC intervention, the depression levels of subthreshold depression adolescents decreased significantly (*F* = 59.482, *p* < 0.001) [18]. The same effect was also found in the elderly after 6-month TCC intervention [19]. These studies have consistently found that TCC is effective for depression throughout the lifespan. Besides, TCC interventions investigated ninety-two prenatally depressed pregnant women for 22 weeks. Their depression (CES-D) scores were significantly reduced [20]. The behavioral evidences greatly supported the positive role of TCC on the decreasing depressive level in nonclinical populations. Moreover, TCC could also ameliorate the depressive level in patients with physical diseases or mental disorders. For instance, a 12-week intervention study with random control trial design reported that the scores on the depression scales decreased significantly after 12-week TCC practice among the old adults with depressive disorder [21]. Some studies lasted for at least 2 months and also showed TCC intervention decreased depression score in patients with Parkinson disease [22], cerebrovascular disease [23], as well as multiple sclerosis [17]. Recently, a meta-analysis study of our group examined the effect size of depressive symptom in 1,159 schizophrenic patients, which showed that patients showed moderately significant effects in favor of mind-body exercise intervention to improve depression (SMD = 0.88; 95% CI 0.63–1.13; *p* < 0.00001) [24].

### 2.2. The Effect of TCC Practice on Anxiety

Anxiety frequently brings about some physical symptoms such as shortness of breath, vertigo, palpitation, digestive problems, or other somatic discomforts. Some studies generally combine anxiety scales along with some physiological measurements including sleep, blood pressure, heart rate, electroencephalogram (EEG), electrocardiogram, and other physiological parameters. Most of the evidence supports the positive effect of TCC on reducing anxiety. A 12-week intervention study investigated the change of anxiety among individuals with normal blood pressure having slight anxious symptoms, which showed both state anxiety and trait anxiety decreased as well as other physiological indices including improvements in blood pressure and blood lipids, relative to the control group [25]. TCC interventions were also effective among patients with moderate anxiety symptoms. For example, a group of middle-aged people, scoring higher than 17 points on Hamilton Depression scale, had significantly decreased anxiety after a 10-week TCC intervention [26]. In addition, TCC was found to play a moderating role in reducing anxiety when combined with other interventions [27]. A recent meta-analysis showed that the TCC practice was moderately to largely significant in improving anxiety in nonclinical population [28], which reliably supported that TCC is a worthy complementary nonpharmacological resource towards anxiety.

### 2.3. The Effect of TCC Practice on Stress

Perceived Stress Scale is a common measurement to evaluate the effect of TCC intervention on stress. In a 15-week interventional study, TCC participants used this scale to examine its beneficial effect, which showed that TCC practitioners had lower stress scores after intervention [29]. Regarding the duration of this effect, an investigation found that the effect of TCC on the stress level lasted for 8 weeks after the intervention [30]. Some studies have used physiological measurements such as salivary cortisol, *α*-amylase, heart rate, and other indices to evaluate the effects of TCC on stress. A study randomly assigned all participants into four groups as TCC, walking, mindfulness, and reading groups and administrated two types of stress tasks [31]. The mental arithmetic task and the IQ test were used to induce cognitive stress, while negative movie clips were used to induce emotional stress. The results showed that the TCC group had a significantly lower adrenaline level compared with the mindfulness group, through the higher noradrenaline level relative to the reading group. Furthermore, all groups had a decreased salivary cortisol level after the intervention [31]. This study highlighted the importance of the research methodology needed to investigate the effect of TCC on stress. Although varied measurements were adopted in the studies investigating the role of TCC practice on stress, the results of most studies are consistent both in the condition of long-term intervention and a single bout of TCC practice.

### 2.4. The Effect of TCC Practice on Positive Emotion

It was consistently found that TCC practice could induce positive emotion. A 15-week TCC training could increase the happiness score in the four-dimensional emotional scale among college students. Moreover, those increased scores in happiness, energy, and peacefulness were associated with the increased mindfulness scores [29]. Another study similarly showed 6-month TCC intervention that improved happiness and life satisfaction among the elderly, in which increased scores were associated with the duration of TCC intervention [19]. TCC practice also enhances positive emotion in patients diagnosed with physical diseases. TCC intervention could not only reduce headache scores among tension-type headache patients but also improve happiness feeling, energy, and mental health [32]. Another investigation showed that TCC decreased scores of 39 symptom-related items and increased happiness scores in Parkinson patients [33]. In addition to application into physical disease, TCC was also adopted as a treatment for brain injury, which showed that the scores of sadness, confusion, anger, nervousness, and fear significantly decreased after 3-week TCC training, while happiness and vigor scores dramatically increased [34].

### 2.5. The Integrative Intervention Combining TCC Practice with Other Interventions

Regarding the effect size, some studies also compared TCC practice with other intervention forms as well as the combination of these interventions. A study focusing on comparing TCC and Pilates showed that both exercises could improve college students' mental health including increased self-efficacy and decreased negative experience after 15-week training, which emphasized that the effect of TCC on positive emotion [35]. An acute intervention study administrated questionnaires to 174 participants randomly being assigned into TCC, yoga, wushu, weightlifting, aerobic dancing, and the musical appreciation group at 5 minutes before, 5 minutes after, and 3 hours after the intervention, which showed that after controlling the baseline level and exercise intensity, the combination of TCC and yoga for 60–75 minutes could significantly induce greater calmness and lowered fatigue and exhaustion [36]. Even an integrative intervention of combining TCC with mindfulness that lasted for only 5 weeks increased middle school students' happiness, calmness and relaxation, sleep quality, and self-consciousness, which suggested TCC could be popularized among adolescents [37]. Recently, TCC practice, as one of the main components of multiple model intervention, increasingly attracts researchers' attention for its beneficial role for alleviating negative emotions and enhancing positive emotions.

## 3. Potential Mechanisms Underlying the Effect of TCC on Improving Emotional Health

**Figure 1:**
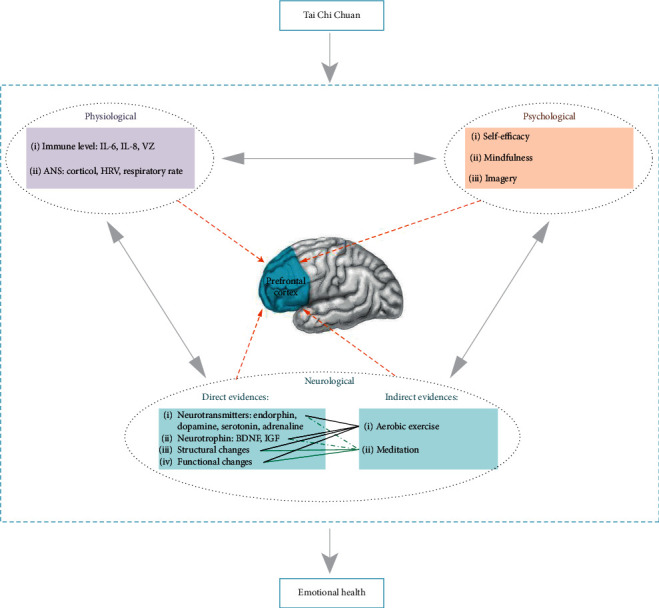
An illustration on multifaceted mechanism of TCC-induced effects on mental health, indicating potential mechanisms from physiological, psychological, and neurological perspectives. The prefrontal cortex hypothesis is proposed based on available direct and indirect research evidence.

Although most of studies reviewed better emotional health following a TCC intervention, it remains largely unknown the mechanisms through which TCC leads to these effects. One reason is that there is no relevant theoretical framework focused on revealing TCC's effect on emotional health. Another reason may be the difficulty of TCC exercise regimen, which may be viewed as a barrier, hindering the ability to evaluate TCC's function in this regard. Here, three potential mechanisms are proposed (Figure 1).

### 3.1. Potential Physiological Mechanisms and Relevant Moderators

It has been suggested that the effect of TCC on emotional health may be related to the change of immune levels. A group of elderly patients taking medication for depression (i.e., escitalopram) had decreased levels of inflammatory markers in C-reactive protein after the TCC intervention [38]. TCC practitioners also had a lower level of interleukin (IL-6), which is associated with decreased depression severity [39]. Researchers have also investigated the relationship between the immune level and emotional health during TCC practice among individuals with chronic health conditions. For example, after 12-week TCC intervention, IL-6, IL-8, and the glucose level increased significantly, which were also associated with decreased anxiety and stress levels in breast cancer patients [40]. Some research evidence indicates that immune levels were increased after 12 weeks of TCC training in both healthy people and diabetic patients, which were also significantly correlated with the clinical symptoms [41, 42]. Similar findings in the cell-mediation immune level of varicella zoster virus demonstrated that the effect of TCC on an individual's immune level could equal that of injecting the varicella vaccine. Additionally, this study also observed that the combination of TCC with varicella zoster virus is far more effective than injecting vaccine only (95% CI = 0.003–0.01; *p* < 0.001). The TCC group had better emotional health after intervention compared to baseline, while the control group had worse emotional health [43]. Accordingly, one potential mechanism is that TCC improves immune levels in the body, leading to better mental health.

Furthermore, it has been hypothesized that the effect of TCC on emotional regulation is closely associated with the autonomic nervous system (ANS) [44]. TCC practice leads to decreased heart rate, which is a biomarker of the sympathetic nervous system [45]. Some studies indicate that the increased salivary cortisol level was associated with increased anxiety [46, 47]. Salivary cortisol is a key hormone controlled by hypothalamic-pituitary-adrenal axis. Evidence has shown that salivary cortisol in TCC groups was lower than other moderate intensity exercise groups or controls [30]. Additionally, heart rate variability (HRV), an important parameter to reflect the activity of the ANS was found to be altered after TCC intervention, because the change maintained the balance of the sympathetic and parasympathetic nervous system and make people's mood more relaxed [48]. Importantly, low frequency and high frequency HRV components are generally considered to be related to sympathetic and parasympathetic responses, while low/high frequency ratio is related to the balance between two. It was observed that low frequency, low/high frequency ratio [49], and total frequency [50] of HRV were greater among TCC practitioners relative to controls at rest [9].

It is well known that irregular respiration is a common symptom among patients with anxiety and panic disorders [51, 52]. The change of the respiratory pattern can be observed when individuals experience a change in emotions such as anger, fear, happiness, and sadness [53]. Respiratory rate was observed to be positively correlated with scores of state anxiety (*r* = 0.756, *p* < 0.05) [54]. A recent study indicated that the sympathetic nervous system during the resting state differed from that of abdominal breathing among experienced TCC practitioners with 21 years of training experience. Specifically, during abdominal breathing, the low frequency, total power frequency, and normalized low frequency components as well as the low-frequency/high-frequency ratio were significantly higher, whereas the normalized high frequency was significantly lower in the TCC practitioners relative to controls during the abdominal breathing state. However, there was no significant difference for these components during the resting breathing state, which provided additional direct physiological evidence for the role of TCC practice in stress reduction [9]. Hence, it is likely that TCC regulates the sympathetic nervous system via abdominal breathing to improve mental health [55, 56].

### 3.2. Potential Psychological Mechanisms and Moderators

Psychological factors are also important variables that influence emotional health during TCC practice. Previous research reported that TCC improved emotional health by improving self-efficacy [57]. Social support was another crucial factor during TCC practice, which enhanced the effect of TCC on mental health [58]. Moreover, mindfulness and imagery are possibly variables that aid in moderating mental health during TCC [59]. TCC is termed as “moving meditation” as it requires quieting the mind while concentrating on slow and gentle movements [59]. An increasing number of research evidence indicates that mindfulness could enhance immune function and improve mood/mental health [60–62] and decrease depression [63]. Hence, the mindfulness component during TCC practice likely contributes to changes in emotional health, as the individual is instructed to quiet the mind and let go of negative emotions [64]. Another unique feature of TCC is imagery. Imagery utilizes multiple pathways including sensation, emotion, and interception to process mental state. It has been consistently observed that imagery decreases depression and enhances positive emotion and life quality [65]. Guided imagery is a general technique to treat mental diseases, such as posttraumatic stress disorder, or social phobia [66, 67]. TCC practice contains various movements following the principle of “the mind guides qi, and the qi operates body” [68]. For instance, “cloud hands,” “white crane spreads its wings,” and “parting the wild horse's mane” are performed while visualizing the movements and leading to less depression [59].

### 3.3. Potential Neurological Mechanisms

There is no direct neurological research evidence to reveal the brain mechanism underlying the TCC on improving emotional health at present. However, researchers provide several potential hypotheses of TCC-induced emotional processing from different perspectives. One emphasizes that TCC could modulate the activity and connectivity of key brain regions or networks involved in emotional disorders such as anxiety and depression [6]. The cognitive control network and default mode network might be altered after practicing TCC for their involvement in attention regulation, self-referential processing, affective cognition, and emotion regulation, in which impairments are typical symptoms of depression [69]. These findings indicate that the central nervous system may play an important role in the modulation effect of TCC. Another valuable hypothesis suggests that the automatic nervous system may also be involved in the modulation effects of TCC on emotional disorders (depression) since accumulating evidences showed TCC practice could optimize the activity of heart rate variability [6, 70].

Although these hypotheses provide insightful viewpoints for future investigation, very few direct evidences to support their role of altering emotional processing or regulation are induced by TCC practice. Therefore, given the complexity of TCC practice involved both in body postures and mind guidance, it is plausible to seek some indirect evidences from the components of TCC such as aerobic exercise and meditation. The average oxygen consumption volume of TCC practice is 55% of maximum oxygen uptake (VO_2_ max) with heart rate at 58% of the optimal heart rate zone [71], so it is regarded as a typical moderate intensity aerobic exercise. In addition, TCC contains meditative components [72, 73], which are an integral part of this mind-body exercise [74], instead the effects of aerobic exercise and meditation training on emotion/mental health provide the underpinning for investigating the effect of TCC on mental health.

#### 3.3.1. Exercise Modulates Brain Function

Most relevant studies have explored the neural correlates underlying the effect of exercise on emotional health by using experimental paradigm-acute exercise. For instance, Pertruzzello and Tate Petruzzello and Tate utilized EEG to investigate the effect of different kinds of exercise intensity on emotions on baseline, 5 minutes, 10 minutes, 20 minutes, and 30 minutes after the intervention. They observed that EEG activity in prefrontal cortex at 5 minutes after intervention contributed to a 23% change in state anxiety, while EEG activity at 25 minutes after intervention resulted in a 22% improvement in overall mental health [75]. Another study used EEG and near-infrared spectroscopy (NIRS) to explore if mood could be changed from 15 minutes of acute exercise (bicycle ergometer). The results found that the blood oxygen level in the ventral prefrontal cortex increased during exercise along with increases in the *α*-wave, while the scores of negative mood decreased and the scores of vigor increased [76]. However, a recent electromagnetic tomography study observed that exercise with maximum intensity significantly worsened negative mood and that *α*- and *β*-activity decreased in the parahippocampus [77].

#### 3.3.2. Exercise Alters Brain Neurotransmitters

It is commonly accepted that endorphins are a key neurotransmitter to regulate emotions during exercise at the molecular level [78]. This hypothesis suggests that the increased positive mood and well-being contribute to releasing endogenous opioids such as beta-endorphins [79]. Endorphin spreads across the central nervous system, which regulates the neuroendocrine system and ANS [80]. It is thought that the opioid peptide is related to positive emotional coping when in a stressful environment [81], while the monoamine hypothesis [82] suggests that exercise prompts releasing neurotransmitters such as dopamine, serotonin, and adrenaline in the brain, which is associated with decreased anxiety and the depression level [83].

#### 3.3.3. Exercise Changes of the Brain-Derived Neurotrophic Factor Level

At the molecular level, the brain-derived neurotrophic factor (BDNF) is another common protein to be associated with decreased depression. Evidence from animal models shows that stress-induced depression changed the BDNF level via cell signal detection [84]. In an electroconvulsive therapy (ECT) study in treating depression, the BDNF level was increased in the hippocampus [85]. Besides, BDNF, Val66Met, and Val/Met were found to be associated with mental disorders in adolescents [84]. Taking together, a lot of animal and human studies have reported that aerobic exercise improves BDNF levels in the brain, increases regeneration in the parahippocampal gyrus [86], and leads to antiaging of the hippocampus [87]. Therefore, TCC likely regulates neuroendocrine cells in key regions relevant to mental health/emotion processing in the brain such as the amygdala, hippocampus, and prefrontal cortex.

#### 3.3.4. Meditation Reshapes Brain Structure and Modulates Brain Function

Neuroimaging studies indicated that long-term meditation increases the cortical thickness in prefrontal and temporal cortex and decreases cortical thickness in the parietal and occipital cortex, as well as altered white matter. These neural processes supported for better sustained attention and emotion [88]. From the perspective of brain structure, it provides the basis for TCC to have a positive impact on human cognitive function. A mindfulness study among generalized anxiety disorder patients reported that activation in prefrontal cortex increased significantly in the mindfulness group compared to the control group. Also, they found that functional connectivity increased between the amygdala and prefrontal cortex. Moreover, it also showed that these changes in brain activation were correlated positively with participants depression scores [89]. Similarly, a study used functional magnetic resonance imaging to examine the mindfulness-induced emotional effect among the patients with bipolar disorder, which found that an 8-week mindfulness intervention enhanced the ability to regulate emotions and increased the blood oxygen level dependent (BOLD) score in the medial prefrontal cortex and posterior parietal cortex [90].

Notably, it was observed that the increased cortical thickness found in TCC experts overlapped with that of mediation experts [91]. Intriguingly, the altered brain anatomical structures in TCC experts were also similar to the findings on aerobic exercise [91, 92]. Although the relationships among TCC, aerobic exercise, and meditation remain unknown, we surmise that these practices likely share some common neural correlates, though this needs to be investigated in the future.

## 4. The Role of Prefrontal Cortex: A Hypothesis

Ample research evidence demonstrates that the prefrontal cortex plays a crucial role in the effect of mind/body training on emotions/mental health, providing support for our hypothesis. Based on the following evidence, it is reasonable to hypothesize that the prefrontal cortex, as a core component of the human brain network, implements its function on mediating emotions via multiple neuroimmunological and neuropsychological pathways.

### 4.1. Direct Research Evidence

Recently, we directly examined the association between brain plasticity and TCC practice, using the newly developed regional homogeneity (ReHo) method of resting-state functional magnetic resonance imaging (R-fMRI) to examine the differences in intrinsic functional architecture between TCC experts and controls. Our results showed that regional homogeneity in some brain regions relevant to low-level sensory motor function was significantly higher among experts and that homogeneity in other regions relevant to high-level cognitive functioning was significantly lower relative to controls. Intriguingly, optimized local functional organization predicted performance gains on the attention network task among TCC practitioners. Another study investigated whether TCC practice could induce cortical structural change [91]. The results indicated that the cortical thickness in several key brain regions (such as the prefrontal cortex) of TCC practitioners differed significantly from that of the controls. These differences were similar to the reported change in brain structure induced by aerobic fitness and meditation. We hypothesized that TCC might share some common neural correlates associated with meditation and aerobic exercise. Using resting fMRI, we characterized dynamic fluctuations of large-scale intrinsic connectivity networks associated with mind-body practice and examined the difference between healthy controls and TCC practitioners. Compared with the control group, the TCC group had significantly decreased fractional amplitude of low frequency fluctuations (fALFF) in the bilateral frontoparietal network, default mode network, and dorsal prefrontal-angular gyri network. Furthermore, we detected significant associations between mind-body practice experience and fALFF in the default mode network as well as the association between cognitive control performance and fALFF in the frontoparietal network [69]. This study provided initial evidence of the large-scale functional connectivity of brain networks associated with mind-body practice, shedding light into neural network changes accompanying intensive mind-body training, and highlighted the function of the frontoparietal network in the context of the “immune system” of mental health, which was recently developed on the basis of the flexible hub theory. In a different 10-month longitudinal study, we found that TCC training decreased the negative mood, which was correlated with functional connectivity between the prefrontal cortex and key regions relevant to emotion, as well as the grey matter in the prefrontal cortex [9].

### 4.2. Indirect Research Evidence

First, current neuroimaging research evidence supports the critical role of prefrontal cortex among individuals participating in aerobic exercise and meditation training. It is reasonable to infer that the aerobic exercise and meditation components of TCC practice play an important role in emotions/mental health processed by the prefrontal cortex. For instance, mindfulness regulates attention via the lateral prefrontal cortex, which affects the typical neural pathways relevant to the negative mood [93]. Importantly, previous brain imaging studies revealed that exercise regulates negative emotions by increasing the activity of the prefrontal cortex. For example, EEG studies observed that acute aerobic exercise reduced nervousness and anxiety, while simultaneously increasing alpha-1 activity in the right prefrontal cortex, with decreased alpha-2 activity in the anterior cingulate cortex [94]. In another study, NIRS was used to examine the effect of acute exercise on emotions. These results indicate that 15 minutes of aerobic exercise can significantly decrease negative emotions, which was associated with greater activation in the ventral prefrontal cortex. [76] Additionally, an fMRI study also observed that 30 minutes of acute exercise improved negative emotion and decreased activation in reward-related cortex [95]. In a different study, both aerobic exercise and meditation resulted in lasting benefits for 8 weeks after the interventions, with decreased negative emotions and increased activation in the ventral prefrontal cortex [96]. This research evidence suggests that aerobic exercise and meditation/mindfulness components during TCC practice likely reduce negative emotions by reshaping anatomical structures and modulating functional activity in the prefrontal cortex.

Second, some research evidence at the molecular level was conducted to address the role of the prefrontal cortex in the regulation of emotions. A growing number of studies have demonstrated that the prefrontal cortex is involved in an emotion-related immunomodulation process within multiple cell molecular levels. For example, in the human brain, human herpesvirus 6 (HHV-6) and the varicella-zoster virus have been identified [97]. In a random control trial, TCC led to the increased cells-mediated immune level, which was also associated with improved mood/mental health [39, 43]. Although there is no direct evidence to reveal the relationship between TCC practice, immune level and brain activity, we speculate that it is likely that TCC could regulate negative emotions via changing the number of immune antibodies in the prefrontal cortex. Additionally, Kuper and his colleagues observed that decreased grey matter in HIV patients were positively correlated with disease course [98]. Furthermore, the decreased grey matter in the occipital cortex was correlated with numbers of lower CD4 (a type of immunosuppressive T lymphocytes) [98]. These results suggest that TCC could increase CD4 levels, which indirectly supports the potential effect of TCC practice on emotion-related immune cells. TCC practice may possibly influence immune response via the prefrontal cortex or some other brain structures connecting with the prefrontal cortex, leading to changes in emotions/mental health.

Third, transmitters related to emotion in the prefrontal cortex likely contribute to the effect of TCC on emotions. Medial prefrontal cortex (mPFC) plays an important role in the HPA axis [99]. For example, increased dopamine in the HPA axis was associated with increased dopamine receptors in the mPFC among pregnant rats exposed to alcohol [97]. Such evidence further supports the role of the prefrontal cortex as a bridge between the central nervous system and the autonomic nervous system along the stress-related neural pathway. The most direct evidence from molecular mechanisms related to negative emotion indicated that opioid-mediated placebo effects greatly reduced pain and negative emotion, which generally regarded as a function of dorsal lateral prefrontal cortex (DLPFC) [100]. Similarly, another study found that when the prelimbic area in the prefrontal cortex (responsible for defensive behavior) was injected with cannabidiol (CBD), rats were performed in the opposite behavior. This CBD effect was described as being mediated by serotonin receptors [101]. Clinical research evidence indicates that continuously decreasing activity in the prefrontal cortex was linked to decreased dopamine levels in patients with schizophrenia. This suggests that there is an association between mental health disorders and altered transmitters in the prefrontal cortex [102].

**Figure 2:**
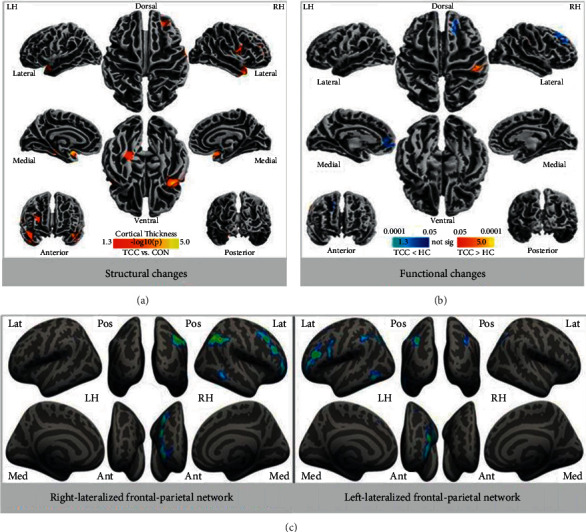
An illustration of direct research evidence from brain imaging of the prefrontal cortex. (a) Structural changes: thicker cortical regions in the TCC group; (b) functional changes: functional homogeneity measured by 2dReHo in the TCC group compared to the control group (blue colors indicate decreases in 2dReHo, while red colors indicate increases in 2dReHo; (c) intrinsic connectivity network based on 12 brain networks, the left panel indicates the right-lateralized frontal-parietal network, while the right panel indicates the left-lateralized frontal-parietal network.

Based on prior research, we hypothesize that the prefrontal cortex is likely a biomarker responsible for the neural mechanisms mediating the effects on emotions/mental health, especially negative emotions (Figure 2).

### 4.3. Limitations

First, there are no standards or consistent principles regarding TCC exercises regimen such as intensity, frequency, and duration [103, 104], which undoubtedly leads to challenges when attempting to make direct comparisons among results of previous studies. The American College of Sports Medicine (ACSM) defined moderate intensity as 60–75% or 50–74% VO_2_ max. Both the Centers for Disease Control and Prevention in the United States and ACSM suggest that 30 minutes of moderate intensity exercise 5 times each week is effective for improving mental health. To date, no TCC study has focused on different dose-effects on regulating emotional health. Second, most of the prior TCC studies examined the TCC's effect on various negative emotions in a single study. Thus, it is unknown if a specific emotion was influenced more by TCC practice. In view of these issues, we were unable to determine the effect size on each emotion and could not provide scientific evidence to guide clinical treatments/practice. Another problem is that multiple interventions were used simultaneously to examine the effect of TCC on positive emotions. Notably, the placebo effect should be considered in some studies that failed to employ a true control group. Third, it is important to pay attention to the duration of the TCC's effect after intervention in longitudinal studies, when examining changes in emotional health. This information could provide meaningful data to support treatments for mental health disorders. Fourth, study samples contained different demographic characteristics including age, health status, diseases types, and personalities, which may have influenced the results obtained. It is important to note that there are significant individual differences in emotional regulation, which may have limited the popularity of TCC practice. Moreover, cognitive style might be another important variable to investigate alongside mental health, as this undoubtedly plays a key role during TCC practice. Finally, there are many TCC styles such as Chen, Yang, Sun, Wu, and other family styles. While the various TCC styles follow the same principles (e.g., movements come from center), differences among these styles still exist such as the speed of motions, the order of poses, the size of the movements, hand orientation, as well as the way in which the movements are performed. However, some studies failed to provide details on TCC style used and its movement characteristics, which might be challenging when exploring its effect on emotional health in order to make recommendations for clinical practice/treatments.

## 5. Conclusion

TCC is a traditional physical exercise with multiple components and has played an important role in alternative, complementary, and integrative medicine for several decades both in the east and west. Although a substantial body of literature has documented the physical health benefits of TCC, researchers are far from understanding its potential mechanisms. Many questions regarding the general effects of TCC on the brain, at the molecular level, and on genetic transcription cascades remained unresolved. Based on some direct and indirect research evidence, several potential mechanisms from physiological, psychological, and neurological perspectives have been put forward in this review study. Importantly, studies with fMRI demonstrated the relevant change of brain anatomy and function mainly on the prefrontal cortex following TCC practice. Notably, a significant activity pattern in the prefrontal cortex among TCC experts was found. Therefore, the effects of TCC on emotional health is likely due to the prefrontal cortex hypothesis, the “immune system of the mind” indicating the role of the prefrontal cortex as a flexible hub in regulating an individual's emotional health. TCC practice may also initiate brain feedback tools including meditation, deep breathing, and exercise to improve mental health. Thus, the prefrontal cortex is likely a key biomarker among the multiple complex neural correlates to aid with emotional conflicts and negative experiences during daily life.

Future studies are needed to confirm these preliminary observations by adopting rigorous methodologies and by further combining multiple measurements, including physical and psychological measurements, along with basic research. It is especially important to adopt brain imaging techniques to investigate the association between the body, mind, and brain within a TCC theoretical framework to investigate mental health/emotional issues. A promising area of research to pursue is the effect of TCC on emotions/mental health from a brain network perspective, especially exploring the anatomical and functional connections between the executive control system (mainly prefrontal cortex) and limbic network (including amygdala, insula, and hippocampal gyrus).

## Supplementary Materials

Supplementary Materials Figure S1. Topic-related annual number of studies. Table S1. Study on the brain mechanism of Tai Chi regulating emotion.
