# Supplementary material for: The Effect of Tai Chi Chuan on Emotional Health: Potential Mechanisms and Prefrontal Cortex Hypothesis
Source: Evid Based Complement Alternat Med. 2021 Apr 30;2021:5549006. doi: 10.1155/2021/5549006 (PMC8110391; doi:10.1155/2021/5549006)
Supplement: Supplementary Materials — Figure S1. Topic-related annual number of studies. Table S1. Study on the brain mechanism of Tai Chi regulating emotion. [file 5549006.f1.docx]

Three English-language databases (PubMed, We of Science, and EBSCO) and two Chinese-language databases (China National Knowledge Infrastructure and Wanfang) were searched from inception until October 2020. To obtain a maximum of relevant studies, we used three groups of keywords: (1) (1) “anxiety” OR “depression” OR “stress” OR “emotion” OR “affect”; (2) “Tai Chi” OR “taiji”. A total of 400 articles were retrieved based on the searching result. We found that the number of publications is dramatically increasing, especially in the last decade (see supfigure 1). Finally, we added “functional MRI” OR “MRI” OR “EEG” OR “ERP” OR “fNIRS” to search the brain imaging studies relevant to this topic. Only 6 articles were retrieved including two reviews.

Figure 1S Topic related annual number of articles

Table 1S Study on the brain mechanism of Tai Chi regulating emotion

| Study | Sample size | Mean age  (SD) | Female | Brain region | Significant findings |
| --- | --- | --- | --- | --- | --- |
| (Yu et al., 2018) | * | * | * | * | the TCC favorable effects on alleviating anxiety, depression and mood disorder in different populations; Prefrontal lobe, insula and cingulate gyrus play a key role . |
| (Port et al., 2018) | 8 | 66.4±4.9 | 5 | superior frontal gyrus; frontal pole; lateral occipital cortex; intracalcarine cortex; occipital pole | Tai Chi group required less brain activation to perform the attention and memory tasks; TC had smaller anxiety. |
| (Gamus, 2015) | * | * | * | * | Tai-chi may reduce balance impairment in mild-to-moderate Parkinson's disease and improve symptoms in patients with osteoarthritis. |
| (Liu, Wu, Li, & Guo, 2018) | 26 | 65.19 ± 2.30 | 18 | dorsolateral  prefrontal cortex；dorsolateral  prefrontal cortex | The impact of the meditative component of tai chi on emotion regulation was mediated by functional connectivity within the executive control network. |
| (Liu et al., 2020) | 31 | 64.94 ± 2.37 | 21 | Precuneus；Cerebelum；Rolandic operculum；Ventral striatum；Middle occipital gyrus；Middle temporal gyrus | long-term Tai Chi exercise may be effective in alleviating feelings of regret in elders by promoting reduced judgment of inner experience and enhanced emotion regulation through the strengthening of fronto-striatal functional connectivity. |
| (Xu et al., 2020) | 16 | 46.5 ± 18.5 | 10 | anterior insula；  posterior insula；  Caudate；  Superior Temporal Gyrus；  Superior Frontal Gyrus；  Superior Parietal Gyrus | differential changes in insula connectivity as neural correlates of symptom improvement in major depressive disorder. |

Gamus, D. (2015). Advances in research of complementary and integrative medicine: a review of recent publications in some of the leading medical journals. *Harefuah, 154*(1), 9-15, 70.

Liu, Z., Li, L., Liu, S., Sun, Y., Li, S., Yi, M., Guo, X. (2020). Reduced feelings of regret and enhanced fronto-striatal connectivity in elders with long-term Tai Chi experience. *Soc Cogn Affect Neurosci, 15*(8), 861-873. doi:10.1093/scan/nsaa111

Liu, Z., Wu, Y., Li, L., & Guo, X. (2018). Functional Connectivity Within the Executive Control Network Mediates the Effects of Long-Term Tai Chi Exercise on Elders' Emotion Regulation. *Front Aging Neurosci, 10*, 315. doi:10.3389/fnagi.2018.00315

Port, A. P., Santaella, D. F., Lacerda, S. S., Speciali, D. S., Balardin, J. B., Lopes, P. B., Kozasa, E. H. (2018). Cognition and brain function in elderly Tai Chi practitioners: A case-control study. *Explore (NY), 14*(5), 352-356. doi:10.1016/j.explore.2018.04.007

Xu, A., Zimmerman, C. S., Lazar, S. W., Ma, Y., Kerr, C. E., & Yeung, A. (2020). Distinct Insular Functional Connectivity Changes Related to Mood and Fatigue Improvements in Major Depressive Disorder Following Tai Chi Training: A Pilot Study. *Front Integr Neurosci, 14*, 25. doi:10.3389/fnint.2020.00025

Yu, A. P., Tam, B. T., Lai, C. W., Yu, D. S., Woo, J., Chung, K. F., Siu, P. M. (2018). Revealing the Neural Mechanisms Underlying the Beneficial Effects of Tai Chi: A Neuroimaging Perspective. *Am J Chin Med, 46*(2), 231-259. doi:10.1142/s0192415x18500131
